# Epidemiological investigation of a foodborne outbreak in Spain associated with U.S. West Coast genotypes of *Vibrio parahaemolyticus*

**DOI:** 10.1186/s40064-016-1728-1

**Published:** 2016-01-27

**Authors:** Jaime Martinez-Urtaza, Andy Powell, Josep Jansa, José Luís Castro Rey, Oscar Paz Montero, Marta García Campello, Mª José Zamora López, Anxela Pousa, Mª José Faraldo Valles, Joaquin Trinanes, Domique Hervio-Heath, William Keay, Amanda Bayley, Rachel Hartnell, Craig Baker-Austin

**Affiliations:** The Milner Centre for Evolution, Department of Biology and Biochemistry, University of Bath, Bath, BA2 7AY UK; Centre for Environment, Fisheries, and Aquaculture Science, Weymouth Laboratory, Weymouth, Dorset DT4 8UB UK; European Centre for Disease Prevention and Control (ECDC), Tomtebodavägen 11 A, 17183 Stockholm, Sweden; Xefatura territorial de Pontevedra, Consellería de Sanidade, Xunta de Galicia, Galicia, Spain; Servizo Microbioloxia, Complexo Hospitalario de Pontevedra, Pontevedra, Spain; Dirección Xeral de Innovación e Xestión da Saúde Pública, Consellería de Sanidade, Xunta de Galicia, Galicia, Spain; Laboratory of Systems, Technological Research Institute, Universidad de Santiago de Compostela, Campus Universitario Sur, Santiago de Compostela, Spain; National Oceanic and Atmospheric Administration, Atlantic Oceanographic and Meteorological Laboratory, 4301 Rickenbacker Causeway, Miami, FL 33149 USA; Rosenstiel School of Marine and Atmospheric Science, University of Miami, Cooperative Institute for Marine and Atmospheric Studies, 4600 Rickenbacker Causeway, Miami, FL 33149 USA; Laboratoire de Microbiologie-LNR, Département Ressources Biologiques et Environnement, Unité Environnement Microbiologie et Phycotoxines, Ifremer, Centre de Brest, ZI de la Pointe du Diable, Plouzané, France

**Keywords:** Seafood, *tdh*, *trh*, *Vibrio parahaemolyticus*, PNW clone, ST 36

## Abstract

We describe an outbreak of seafood-associated *Vibrio parahaemolyticus* in Galicia, Spain in on 18th of August 2012 affecting 100 of the 114 passengers travelling on a food banquet cruise boat. Epidemiological information from 65 people was available from follow-on interviews, of which 51 cases showed symptoms of illness. The food items identified through the questionnaires as the most probable source of the infections was shrimp. This product was unique in showing a statistically significant and the highest OR with a value of 7.59 (1.52–37.71). All the nine strains isolated from stool samples were identified as *V. parahaemolyticus*, seven were positive for both virulence markers *tdh* and *trh*, a single strain was positive for *trh* only and the remaining strain tested negative for both *trh* and *tdh*. This is the largest foodborne *Vibrio* outbreak reported in Europe linked to domestically processed seafood. Moreover, this is the first instance of strains possessing both *tdh*+ and *trh*+ being implicated in an outbreak in Europe and that a combination of strains represent several pathogenicity groups and belonging to different genetic variants were isolated from a single outbreak. Clinical isolates were associated with a novel genetic variant of *V. parahaemolyticus* never detected before in Europe. Further analyses demonstrated that the outbreak isolates showed indistinguishable genetic profiles with hyper-virulent strains from the Pacific Northwest, USA, suggesting a recent transcontinental spread of these strains.

## Background

*Vibrio parahaemolyticus* is a Gram-negative, halophilic bacterium that occurs naturally in estuarine environments worldwide (Kaneko and Colwell 1975). Globally, *V. parahaemolyticus* is the leading cause of bacterial gastroenteritis associated with the consumption of seafood produce (Powell et al. 2013). Potentially virulent strains are most frequently distinguished from likely avirulent strains by the detection of the thermostable direct (*tdh*) and tdh-related (*trh*) hemolysin genes (Bej et al. 1999; Shirai et al. 1990). *V. parahaemolyticus* is the leading cause of seafood-associated bacterial gastroenteritis in the United States (Anonymous 2005; DePaola et al. 2000), and is one of the most important food-borne pathogens in Asia, causing approximately half of the foodborne outbreaks in Southeast Asian countries (Martinez-Urtaza et al. 2004). The number of *V. parahaemolyticus* infections has increased globally during recent years (Nair et al. 2007).

Gastroenteritis caused by *V. parahaemolyticus* has been rarely reported in European countries (Baker-Austin et al. 2010). However, recent studies indicate that the number of *V. parahaemolyticus* infections also appear to be increasing in Europe (Baker-Austin et al. 2010). Currently, vibriosis is not a notifiable disease in Europe (Baker-Austin et al. 2010). Outbreaks of *V. parahaemolyticus* have been regularly reported since 1999, with several outbreaks affecting the Northwest region of Spain. In summer of 1999, an outbreak of *V. parahaemolyticus* affecting 64 persons associated with oyster consumption was detected in the city of Vigo (Martinez-Urtaza et al. 2004). All the strains isolated from stool samples were serotype O4:K11 and belonged to one single and distinctive clone endemic to Europe. A second large outbreak was detected in July 2004 in the city of A Coruña with 80 cases, all of them linked this time to pandcmic O3:K6 strains (Martinez-Urtaza et al. 2005). All clinical cases reported to date in Spain have been exclusively linked to *tdh*-positive and *trh*-negative strains (Baker-Austin et al. 2010). Conversely, large *V. parahaemolyticus* outbreaks have been rarely reported in Europe, with most cases attributed to sporadic cases across Europe.

In the last two decades, several largescale foodborne *V. parahaemolyticus* outbreaks have been reported in the Pacific Northwest region of the United States (Turner et al. 2013; Paranjpye et al. 2012). These bacteria, subsequently termed the Pacific Northwest complex (Turner et al. 2013), appear to be genetically and biochemically distinct, and carry a lower significantly lower infectious dose compared to other toxigenic *V. parahaemolyticus* isolates. Until 2012, infections associated with these bacteria were only reported in the Pacific Northwest region, however, in 2012, infections were subsequently reported along the NE coastline of the USA (Newton et al. 2014; Martinez-Urtaza et al. 2013). We describe here epidemiological and microbiological investigations associated with a large *V. parahaemolyticus* outbreak to identify the source of infections and the most probable origin of the bacteria. This outbreak is particularly noteworthy in that it represents, to our knowledge, the first report of a highly virulent and hitherto Pacific-associated *Vibrio* complex emerging in Europe.

## Methods

### Outbreak setting

On August 19, 2012, the Public Health Department of Galicia, Spain, was alerted to a possible outbreak of illness in O Grove (Pontevedra, NW Spain). All the cases were passengers travelling on a tourist cruise boat that included a visit to the rias (coastal inlets) followed by a dinner. At the time of the reported outbreak, warm weather conditions prevailed in this region of Galicia, with average air temperatures exceeding 25 °C.

### Environmental investigation

Only nine cases were subjected to stool analysis. No left over food was available for analysis. The restaurant and origin of all the food consumed on the cruise were subsequently investigated. All the food consumed on the cruise was traced to its harvest site by the dealer records. The facility where the different items were stored and the premises used for processing and handling the prior the shipment to the restaurant were investigated, and the processing practices were also reviewed to assess the factors contributing to contamination, survival and proliferation of potentially causative microorganisms. Samples of raw product belonging to the same batch of suspected vehicle of infection were subjected to microbiological analyses.

### Epidemiological investigation

Travellers were interviewed using epidemiological questionnaires instigated by the Regional Public Health Department (Conselleria de Sanidade, Xunta de Galicia). Questionnaires gathered a range of data, including age, gender, food consumption history, and date and type of symptoms. A case was defined as a person who consumed food during the cruise and presented with vomiting and/or diarrhea symptoms within 96 h. While a control was defined as a person who consumed food during the cruise and did not present with vomiting and/or diarrhea within 96 h. The restaurant staffs were also interviewed. Cases were defined as individuals who had dinner on the boat on Saturday 18th August 2013, and who reported symptoms of diarrhoea and/or vomiting during the following 72 h after the dinner (see above). A total of 54 persons belonging to the organized group and other 11 additional persons attending the dinner were identified. Epidemiological information from 65 people was subsequently available from follow-on interviews, of which 51 cases showed symptoms of illness and the remaining 14 people did not report associated symptoms. Travellers were interviewed using epidemiological questionnaires by the Regional Public Health Department (Conselleria de Sanidade, Xunta de Galicia). All the subject gave permission to use the questionnaire data for research publication.

### Microbiological and molecular investigation

The local authority in Galicia test suspected gastrointestinal cases during the season of highest risk for Vibrio infections (April–August) using TCBSas an additional microbiological plating media. Stool specimens were collected from patients during the illness outbreaks and plated on Trypticase Soy Agar with 5 % sheep blood and McConkey Agar (Becton–Dickinson, Sparks, MD), and incubated at 37 °C for 18–24 h. Lactose negative colonies on McConkey Agar and/or beta-haemolytic and oxidase positive colonies on Trypticase Soy Blood Agar, were selected and subjected to species identification by biochemical tests on API 20E strips (BioMérieux, Marcy-l’Etoile, France). Serotyping. Lipopolysaccharide (O) and capsular (K) serotypes were determined by agglutination tests using specific antisera, essentially as described previously (Martinez-Urtaza et al. 2004). Presumptive identification of the isolates was confirmed by the presence of the species-specific *toxR* and *tlh* genes. The presence of the *toxR* gene was investigated by PCR as described previously (Kim et al. 1999). Additionally, presence of *tlh*, *tdh* and *trh* genes was determined by multiplex PCR according to the procedure described by Bej et al. (Bej et al. 1999). A GS-PCR assay to specifically detect the pandemic clone-specific nucleotide sequence in the *toxRS* operon of *V. parahaemolyticus* was also performed as described previously (Okuda et al. 1997). Isolates were tested against a battery of antibiotics.

Pulse Field Gel Electrophoresis (PFGE) was performed according to the “One-Day (24–28 h) Standardized Laboratory Protocol for Molecular Subtyping of Non-typhoidal Salmonella by PFGE” (Pulse-Net, CDC, Atlanta, USA) (Anonymous 2002) following a method described previously (Martinez-Urtaza et al. 2004). Chromosomal DNA was digested with 30 U of NotI (Promega, Southampton, United Kingdom) at 37° for 4 h. DNA macro-restriction fragments were resolved on 1 % SeaKem Gold Agarose (Cambrex, Baltimore, MD) in 0.5X TBE buffer. DNA from *Salmonella* Braenderup H9812 restricted with 50 U of XbaI (Promega, Madison, WI) at 37º for two h was used as a size marker. Pulse times were ramped from two to 40 s during a 18-h run at 6.0 V/cm. Restriction patterns were compared with the use of BioNumerics software (Applied Maths). Multi-locus sequence typing (MLST) analysis was performed as previously described (Gonzalez-Escalona et al. 2008) based on internal fragments of seven housekeeping genes: *recA*, *gyrB*, *dnaE*, *dtdS*, *pntA*, *pyrC*, and *tnaA*. Sequences of both strands were determined by custom sequencing (Macrogen Inc., Seoul, South Korea). All chromatograms were assembled, manually edited and trimmed in Bionumerics 5.1 (Applied-Maths, Kortrijk, Belgium). Allele numbers were assigned to each isolate by comparing the nucleotide sequence at each locus to all known corresponding alleles available at the *V. parahaemolyticus* MLST Database (http://pubmlst.org/vparahaemolyticus/).

### Environmental and epidemiological surveillance

Following the Galician 2012 outbreak, several sentinel sites in NW Europe (2 along the southern coast of the UK, and 2 in Northern France) were selected to ascertain if this new *V. parahaemolyticus* variant had emerged in the region. The sites were chosen because they encompass commercially important shellfish harvesting areas that could be analysed practically by participating laboratories over a prolonged period of time in NW Europe. Bivalve shellfish produce monthly (Pacific oysters and Common Mussels), from the Autumn of 2012 until the Winter of 2015, were tested for *V. parahaemolyticus* in the facilities of IFREMER (France) and CEFAS (UK). Bivalve shellfish produce was processed and tested using molecular methods essentially as previously described (Powell et al. 2013). Strains that were identified possessing the virulence markers *tdh* and *trh* were subsequently screened using subtyping methods such as MLST and serotyping. Alongside field-based surveillance, we also contacted relevant health authorities as well as reference laboratories in Europe to identify any clinical cases of *V. parahaemolyticus* linked to the new variant identified from member state investigations.

## Results

On 19th of August 2012, an outbreak of gastroenteritis was reported among passengers travelling the previous day on a food banquet cruise boat near Pontevedra, Galicia, NW Spain. The trip included a visit to the rias (coastal inlets) followed by a dinner mostly comprising locally harvested and imported seafood produce. A total of 114 individuals attended the dinner, 54 of them belonging to an organized group. The food items identified through the questionnaires as the most probable vehicle of the infections was shrimp (Table 1). This product was unique in showing a statistically significant (*p* = 0.006) and the highest RR [2.19 (0.98–5.40)]. Shrimp (frozen) served at the banquet, were processed with a short boiling of 1–2 min, and once cooked, were submerged in water with ice for a rapid cooling. Follow-on investigations indicated that the ice (and potentially water) employed to cool seafood produce was in contact with mussels and cockles obtained from a local authorized shellfish purification plants until they were boiled. Preliminary analyses showed that 51 cases were detected among the 65 persons available who had dinner on the cruise boat, which represents a 78 % attack rate associated with this outbreak. The average incubation period was 14 h, with a minimum of 2 h and maximum of 57 h since the detection of symptoms. The disease symptoms were: diarrhoea (98 %), abdominal pain (51 %), vomiting (33 %), nausea (24 %), low-grade fever (18 %) and fever (2 %). The mean duration of symptoms was 56 h, with a minimum of 10 h and maximum of 259 h (10 days and 19 h). From the total of cases, 28 cases subsequently required medical attention.

**Table 1:** Epidemiological analysis of food items implicated in outbreak based on questionnaires

Food	Consumed food item			Did not consume food item			Statistical analysis		
	Ill	Not ill	Total	Ill	Not ill	Total	Relative risk	95 % CI	*p* value
Galician pie	34	9	43	10	5	15	0.97	0.72–1.30	0.844
Norway lobster	21	6	27	23	8	31	1.09	0.81–1.48	0.550
Small crabs	26	7	33	18	7	25	1.05	0.78–1.40	0.750
Shrimp	*41*	*9*	*50*	*3*	*5*	*8*	*2.19*	*0.89*–*5.40*	*0.006*
Cockles	31	8	39	13	6	19	1.16	0.82–1.64	0.355
Mussels	31	8	39	13	6	19	1.16	0.82–1.64	0.355
Rice with seafood	7	0	7	37	14	51	1.38	1.16–1.63	0.112
Beef	3	2	5	41	12	53	0.78	0.37–1.61	0.386
Cake	37	12	49	6	1	7	0.87	0.62–1.22	0.516

*p* value < 0.05 are in italics

Nine stool samples were collected and subjected to analysis at a local hospital (Complejo Hospitalario de Pontevedra). All the nine cultures were positive for *V. parahaemolyticus* and subject to further characterisation at the Centre for Environment, Fisheries and Aquaculture Science (CEFAS, UK). All strains tested positive for *toxR* and *tlh*, confirming that all isolates were *V. parahaemolyticus.* Seven of the nine strains were *tdh* and *trh* positive, with a single isolated strain positive for *trh* only and another strains being negative for both hemolysin genes. All the isolates were susceptible to doxycycline and ciprofloxacin, and resistant to ampicillin and 1st generation cephalosporins.

**Fig. 1:**
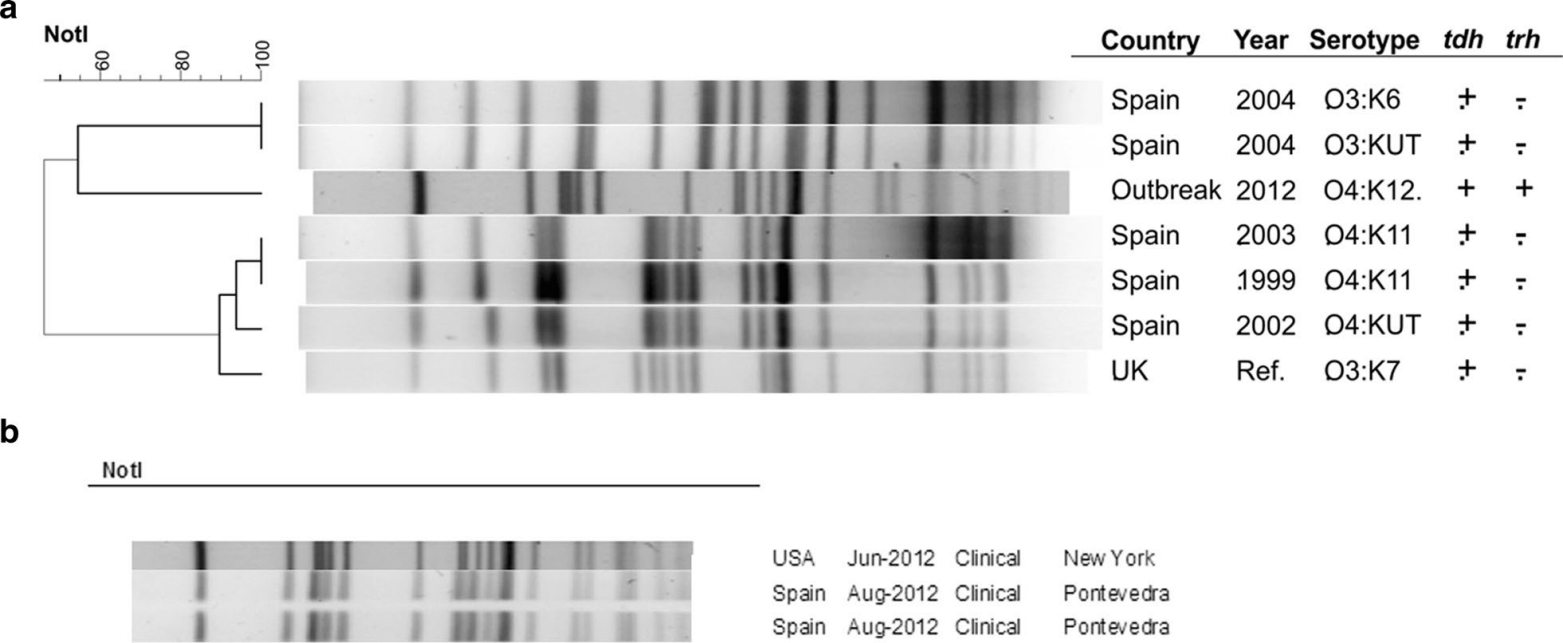
PFGE analysis of the outbreak strains. **a** PFGE profiles of all available clinical strains of *V. parahaemolycus* detected in Spain since 1998, including one reference strains from clinical sources isolated in the UK, and PFGE profile of outbreak strains. **b** PFGE of *V. parahaemolyticus* strains from New York outbreak (June 2012) and Pontevedra, NW Spain, August 2012 (PFGE data from New York strain courtesy Gladys Gonzalez-Aviles, CDC, USA)

**Fig. 2:**
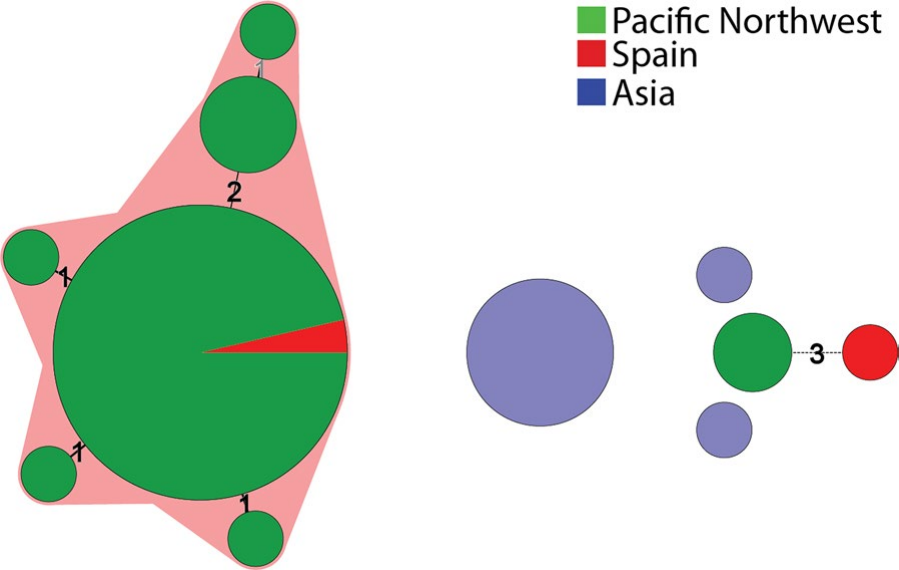
Genotyping analysis of the two genetic variants isolated from the outbreak. Minimum Spanning Tree based on MLST alleles constructed using categorical coefficient with the tdh+/trh+ outbreak strain includes into the ST 36 group with strains characteristic of the Pacific Northwest of North America and the second outbreak strain tdh-/trh+ with a novel ST uniquely associated with ST 417 strains, other genetic variant endemic for the Pacific NW of North America; *each circle* represent a different ST, the *size of the circle* reflects the number of isolates, *numbers in branches* indicate the number of loci with different alleles and orange areas designate clonal complex by Bionumerics grouping genotypes differing in a maximum of two loci

All recovered isolates were serotyped using commercially available *V. parahaemolyticus* antisera and subsequently subjected to pulsed field gel electrophoresis (PFGE) and multi locus sequence typing (MLST) analyses. With the aim of identifying a common origin of the outbreak strains, Spanish outbreak *V. parahaemolyticus* isolates were subsequently analysed by MLST and results were compared with data relative to clinical and environmental strains included in the MLST database (http://pubmlst.org/vparahaemolyticus/). PFGE analysis identified the Galician outbreak strains as a novel group hitherto unidentified in Europe, and different from PFGE from strains previously isolated from clinical and environmental sources in the region (Fig. 1a). Comparison of Galician strains with PFGE data of strains from a recent outbreak in New York two months before (Newton et al. 2014) showed that both outbreak strains shared indistinguishable PFGE profiles (Fig. 1b). MLST analysis revealed the existence of two different genetic variants among the outbreak strains. Allele profiles of *tdh* and *trh* positive strains matched with the ST 36, the dominant MLST type in the Pacific NW of North America. Strain *tdh* negative and *trh* positive shared the allelic profiles of six genes with ST 417, also endemic in the Pacific NW, although it showed a new allelic variant for *pyrC* gene according to the MLST database (Fig. 2). This finding was also supported by the serotype analysis which identified the outbreak serotypes were O4:K12 and O4:Kut, dominant groups in the Pacific NW of the USA. The other isolate identified as tdh−/trh+ was untypable by serotyping, whereas the tdh−/trh− isolate was O4:KUntypable. Both isolates showed a new combinations of allelic profiles and were assigned to the two new MLST types ST1031 (tdh−/trh+) and ST1032 (tdh−/trh−), both unrelated to the ST36.

Following the outbreak in 2012, shellfish samples (oysters and mussels) were obtained from several sites along the NW coast of France and Southwest coast of the UK as part of ad hoc testing to identify the potential emergence of ST36 strains into Europe. Samples were collected from commercial shellfish harvesting areas and tested monthly. Of the several hundred *V. parahaemolyticus* isolates obtained from the samples and subjected to characterisation, only one *tdh*+/*trh*+ strain was identified in the southwest of the UK in 2014, however, this strain did not either belong to the same serotype (O4:K12) or MLST type (ST36) previously associated with infections in Galicia.

## Discussion

Several factors make this a highly unusual and noteworthy outbreak. Firstly, this is the first instance, to our knowledge, of strains possessing both *tdh*+ and *trh*+ being implicated in an outbreak in Europe. Secondly, the strains identified from preliminary analyses represent several pathogenicity groups, including strains possessing a mixture of hemolysin genes combinations and different STs and pulsotypes. Similar strains possessing these genes have been identified as causing disease in the PNW of the USA (Paranjpye et al. 2012; Newton et al. 2014), and a highly noteworthy characteristic of these *tdh*+ and *trh*+ is a significantly higher attack rate than other variants of *V. parahaemolyticus* (Martinez-Urtaza et al. 2010; McLaughlin et al. 2005). Taken together, these finding suggest that a new and highly virulent strain of *V. parahaemolyticus* has emerged in Europe. Finally, results from subtyping analyses (including PFGE, MLST, and serotyping) additionally point out that the outbreak isolates closely related, and that these populations were detected in NY 2 months before the outbreak in Galicia (Newton et al. 2014).

**Fig. 3:**
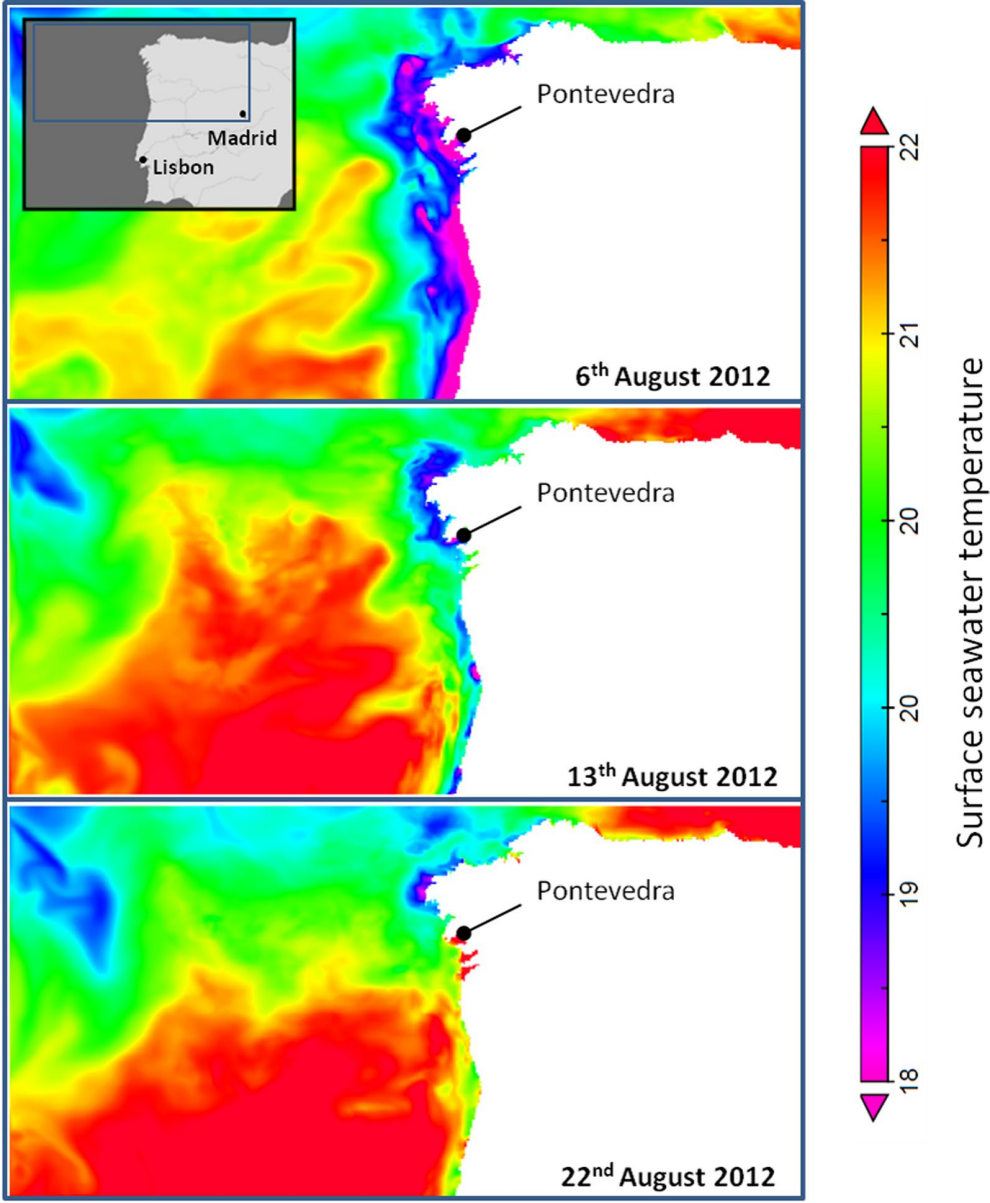
Surface seawater temperatures in North West Spain, August 2012. Note significant warming trend in Pontevedra area corresponding temporally and spatially with incident of outbreak report on 18th August 2012

Based on a retrospective epidemiological analysis of seafood produce consumed in the dinner (Table 1), shrimp showed the strongest association with cases. From an epidemiological perspective, the origin and nature of the shrimp produce (frozen wild animals from the continental shelf in Argentina) made this product an unlikely direct source of contamination. Additionally, subsequent analyses of frozen shrimp were not able to identify pathogenic *V. parahaemolyticus* strains. However, epidemiological investigations through questionnaires and food consumption by the different groups unambiguously identified this product as the most probable source of infection. According to the investigation, it is very plausible that pathogenic *V. parahaemolyticus* strains emerged via cross-contamination through the ice and water used after initial cooking. The ice employed to cool down the shrimp was in contact with local moluscan shellfish that may be the source of pathogenic strains. As the product was hot after boiling and stored at room temperature, the warm conditions may have promoted rapid bacterial growth until reaching levels likely to initiate infections. This hypothesis is further supported by the identification of anomalously warm water in the local region prior to, and during the outbreak (Fig. 3). The incursion of anomalously warm waters from the ocean in the rias has previously been associated with *V. parahaemolyticus* infections in Galicia (Baker-Austin et al. 2010). A noteworthy characteristic of this outbreak is that several strains were recovered from clinical sources, an unusual feature and uncommon during *V. parahaemolyticus* foodborne incidents. This is a striking feature of the outbreak that should be the subject of further investigation, as it implies potential cross-contamination of shellfish produce from several possible sources.

The detection in Galicia of three of the major *V. parahaemolyticus* outbreaks in Europe over the last 15 years stresses the importance of the existence of surveillance programs to enable the detection of *V. parahaemolyticus* infections. The regional government of Galicia (Xunta de Galicia) has been a strong advocate in the declaration of *V. parahaemolyticus* infections as notifiable from 1995 onwards, and this study underlines the growing importance of this pathogen as a serious cause of disease. We were fortunate in this instance that some strains were available for molecular and serological analysis, as in Europe there are significant gaps in laboratory testing, monitoring and reporting of such pathogens, which often precludes the capture of strains. The extension of surveillance programs to other areas in Europe with significant shellfish production and consumption may provide an important contribution to achieve a more accurate estimate of risk of *Vibrio* infections and the burden of this disease in Europe. The potential cross-contamination of shellfish produce using ice should be considered a risk activity in terms of *Vibrio* contamination. From an environmental perspective, the source of the strains and the mechanistic basis of long distance dispersion into Europe still remain unresolved. The absence of systematic programs for monitoring pathogenic *Vibrio* in shellfish harvesting areas in European countries represents a serious limitation to detect new local or alien pathogenic variants. In this regard, we have continued ad hoc surveillance along the Atlantic coast during 2013 and through 2014. Environmental monitoring carried out in some areas of the NW Europe to identify the presence of ST36 strains emerging in the area for the two seasons of highest risk following the outbreak (summer 2013 and summer 2014) was unsuccessful in identifying ST36 or O4:K14/O4:Kut isolates. Subsequent ad hoc collaborations with different microbiology reference laboratories in Europe since the 2012 outbreak have not identified U.S. Pacific Northwest strains residing in Europe, or causing infections. Although no further outbreaks of *V. parahaemolyticu*s have been reported in Galicia linked to these strains, it remains unclear if these highly pathogenic variants have persisted in the region due to the absence of regular environmental monitoring of *V. parahaemolyticus* in the region, as they appear to have along the Eastern seaboard of the Atlantic ocean (Newton et al. 2014; Martinez-Urtaza et al. 2013). Recent data from the USA suggests that these highly pathogenic strains are possibly becoming endemic in an expanding area of the Atlantic Ocean (Newton et al. 2014), with the potential to substantially increase disease risk. Taken together, the link between these and *V. parahaemolyticus* strains from the PNW implicates trans-oceanic movement of these pathogens in disease emergence. A key area of future work will entail determining the precise mechanistic basis of this long distance transport as well as ascertaining the prevalence of these strains in other foodborne outbreaks.

## Conclusion

The outbreak describe here represents a second event of illness related to the introduction of a foreign variant of *V. parahaemolyticus* in the same region. The first instance was reported in 2004 during an outbreak associated with the pandemic clone of *V. parahaemolyticus*. This situation stresses the necessity of designing and implementing new programs for enhancing the control and monitoring of seafood products in regions such as Galicia associated with the risk of introduction of foreign variants of pathogenic vibrios in natural environment and the consequent risk of dissemination into Europe. In particular, the data presented here regarding *V. parahaemolyticus* infections in Galicia underpins the critical relevance of including these pathogens among the notifiable diseases in regions characterised by high levels of shellfish consumption. Finally, the development of a centralised and harmonised monitoring, surveillance and reporting system in Europe would contribute to provide understanding of the real risk of Vibrio infections in Europe, as well as to understand future trends in the risks in a context of warming coastal areas.
